# Child health challenges in the 2025 Sumatra flood and landslide: Lessons for disaster risk reduction and emergency response

**DOI:** 10.4102/jamba.v18i1.2105

**Published:** 2026-10-16

**Authors:** Kurniawan T. Kadafi, Dimas D. Saputro, Martinus M. Leman, Aslinar Aslinar, Badai B. Nasution, Dimas T. Anantyo, Niken W. Puspaningtyas, Fathy Z. Pohan, Angga Wirahmadi, I. Nyoman A. Purwana, Muhammad Reza, Mohamed A. Bin Hj Abdul Rahman, Jaya A. Effendi, Yogi Prawira, Rizky Adriansyah, Raihan Raihan, Asrawati Nurdin, Piprim B. Yanuarso

**Affiliations:** 1 Department of Child Health, Faculty of Medicine, Universitas Brawijaya, Malang, Indonesia; 2 Department of Child Health, Saiful Anwar General Hospital, Malang, Indonesia; 3 Disaster Task Force, Indonesian Pediatric Society, Jakarta, Indonesia; 4 Department of Child Health, Harapan Kita Women and Children Hospital, Jakarta, Indonesia; 5 Department of Paediatric, Faculty of Medicine, Gunadarma University, Depok, Indonesia; 6 Department of Pediatric, Faculty of Medicine, Universitas Sumatera Utara, Medan, Indonesia; 7 Department of Pediatric, Faculty of Medicine, Universitas Diponegoro, Semarang, Indonesia; 8 Department of Child Health, Faculty of Medicine, Universitas Indonesia, Jakarta, Indonesia; 9 Jantung Pembuluh Darah Harapan Kita Hospital, Jakarta, Indonesia; 10 Department of Emergency Medicine, Hospital Selayang, Selangor, Malaysia; 11 Department of Paediatric, Fatmawati Hospital, Jakarta, Indonesia; 12 Department of Paediatric, Zainoel Abidin Hospital, Banda Aceh, Indonesia; 13 Department of Paediatric, Faculty of Medicine, Universitas Andalas, Padang, Indonesia

**Keywords:** Sumatra, flooding, landslide, disaster, children, physical impacts, psychosocial impacts

## Abstract

In late 2025, catastrophic floods and landslides struck Sumatra, Indonesia, causing widespread destruction and nearly 1200 fatalities. Children were among the most severely affected, experiencing acute health problems, disruptions in essential healthcare services and significant psychosocial distress. This essay employs an evidence-informed analytical approach, integrating contemporaneous official reports, available scientific literature, international disaster risk reduction (DRR) frameworks, Emergency Medical Team (EMT) standards and the authors’ direct field experience during the disaster response. The disaster is examined across the disaster management cycle, including prevention, mitigation, preparedness, emergency response and recovery, to identify gaps between established child-centred disaster management principles and their practical implementation. The analysis demonstrates that, despite national policies and international guidance, implementation was inconsistent. Many children experienced preventable illnesses, interruptions in chronic medical care, inadequate continuity of paediatric healthcare and unmet psychosocial needs because of coordination challenges, limited child-focused preparedness and fragmented service delivery. The findings highlight that integrating child-centred DRR principles with specialised paediatric emergency training, strengthened EMT deployment, continuity of essential child health services, coordinated mental health support and multisectoral collaboration can improve disaster preparedness and emergency response. The lessons learned provide practical guidance for strengthening child-centred DRR, improving emergency response systems and informing policy development that better integrates child protection and paediatric healthcare into all phases of disaster management.

## Introduction

The flood disaster in Indonesia occurred in late 2025 and early 2026 on the island of Sumatra. The disaster affected three provinces: Aceh, North Sumatra and West Sumatra. Disasters, whether natural or man-made, pose severe challenges to health systems, with children among the most vulnerable populations (Onyejesi et al. 2025). The 2025 Sumatra flood and landslide disaster exemplified this vulnerability. Torrential rains (exacerbated by deforestation and a rare cyclone) triggered massive flooding and landslides across Aceh, North Sumatra and West Sumatra provinces (Nasri et al. 2025). The geographical distribution of the affected areas is presented in Figure 1. By January 2026, The Indonesia National Disaster Management Agency reported 1200 people killed and over 195 000 displaced (Badan Nasional Penanggulangan Bencana 2026). Among those affected, many children suffered injuries from being pierced by wooden debris, infectious diseases and psychosocial trauma. Children are not simply ‘small adults’ – their developing bodies and minds make them especially susceptible to disasters. For instance, children have smaller blood volumes and faster metabolisms, which can lead to rapid dehydration or shock, and they rely on caregivers for protection and emotional support. These factors exacerbate their risk of illness, injury and psychological harm in crises (Kadafi et al. 2023). Globally, nearly 1 billion children live in disaster-prone countries, and when disasters strike, youths face myriad health challenges such as malnutrition, diarrhoeal disease and respiratory illnesses, along with heightened risks of anxiety, depression and post-traumatic stress (Kadafi et al. 2022, 2023).

**FIGURE 1:**
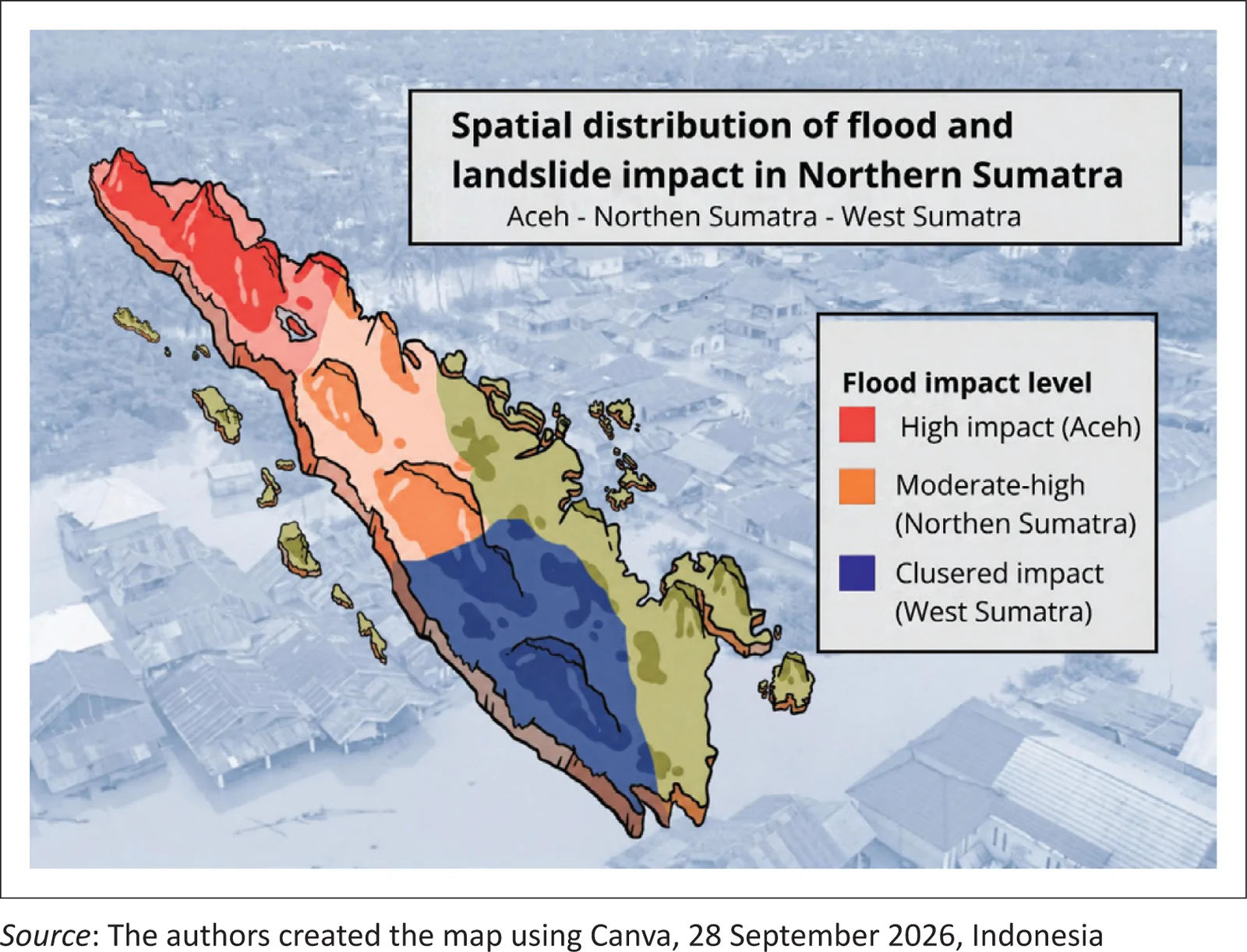
A map illustrating the specific districts affected by flood-related damage.

This article examines the Sumatra disaster through a child-health lens, comparing the *ideal* frameworks for disaster management with the *reality* of the response on the ground. The discussion is organised around the disaster management cycle – preparedness, response and recovery – highlighting how each phase addressed (or overlooked) children’s health needs. We draw on international guidelines (including the Sendai Framework’s emphasis on vulnerable groups and the World Health Organization’s Emergency Medical Teams [EMTs] standards) and Indonesian paediatric disaster preparedness initiatives to frame our analysis. The goal is to derive insights and lessons that have implications for theory, practice and policy in disaster risk reduction (DRR), child protection and health emergency response. By exploring what worked and what fell short in Sumatra, we seek to inform more resilient and child-centred approaches to future disasters.

## Discussion

### Pre-disaster preparedness and risk reduction for children

Before disaster strikes, mitigation and preparedness measures can greatly reduce risks to children. Indonesia has developed various DRR frameworks and educational programmes intended to protect its most vulnerable. The Indonesia National Disaster Management Agency publishes guidelines such as the *Tanggap Tangkas Tangguh* pocket book on disaster readiness (2012) and, in collaboration with the Ministry of Education, a *Safe Schools* curriculum to inculcate a culture of safety (Badan Nasional Penanggulangan Bencana 2012; Koswara et al. 2019). Notably, contingency planning efforts at local levels have begun to adopt a child-centred approach. A United Nations International Children’s Emergency Fund (UNICEF)-supported initiative helped strengthen local contingency plans focused on children’s needs, asserting that planning must recognise children’s rights to survival and protection and ensure their participation in DRR activities (Wiryaningsih et al. 2022). This inclusive approach treats youths as agents of DRR, not just passive victims, by involving them (appropriately) in preparedness efforts. Such initiatives align with global DRR theory that emphasises community and child participation as key to building resilience (DiMaio 2024).

In practice, several disaster mitigation measures related to child health issues had been put in place prior to the 2025 floods in Sumatra. The Indonesian Pediatric Society had a Disaster Task Force that produced a paediatric disaster management handbook in 2023 (Satuan Tugas Penanggulangan Bencana 2023). This guide provides paediatricians and general doctors with protocols for children’s health in emergencies, covering volunteer registration, management of likely paediatric illnesses during disasters and maintaining immunisations (Satuan Tugas Penanggulangan Bencana 2023). Training programmes were conducted to prepare health workers: Hospital mass-casualty drills, Basic Life Support and trauma management courses and the formation of EMTs, multidisciplinary health volunteer teams ready to deploy to disasters (Kang, Pek & Venkataraman 2021). The Ministry of Health, through its Crisis Centre, maintained contingency plans for various disaster scenarios, outlining referral systems and early disease surveillance, and it pre-positioned medical stockpiles (emergency medicines, vaccines, etc.) to enable a rapid health response. These measures aimed to ensure that when disaster struck, medical aid for children could be delivered quickly and in an organised manner (Pusat Krisis Kesehatan Kementrian Kesehatan Republik Indonesia 2023).

Crucially, Indonesia had also been developing its capacity to field internationally standardised EMTs. By 2025, non-governmental organisations (NGOs) were complementing government efforts, for example, the Muhammadiyah charitable organization established a mobile Type 1 EMT (providing outpatient emergency care) that achieved World Health Organization verification. In October 2025, just weeks before the Sumatra floods, the Muhammadiyah team became Indonesia’s first WHO-certified EMT (International Search and Rescue Germany 2025). This validation confirmed that the team met WHO’s minimum standards (including self-sufficiency and quality of care) for disaster response. Such developments indicate growing practical readiness to address health needs, including those of children, in emergencies. Indonesian Pediatric Society itself trained paediatric specialists across the country in disaster response, encouraging them to join EMTs or specialist teams focused on children’s health (Satuan Tugas Penanggulangan Bencana 2023).

Despite these ideal measures on article, significant gaps remained in preparedness, especially in reaching communities and addressing child-specific vulnerabilities. Public education and socialisation of DRR materials had not penetrated all at-risk areas. Many families and even local educators were unsure how to protect children when the floods hit. One analysis found that disaster media and guidance had not been optimally disseminated, contributing to public confusion during the emergency. Likewise, while some early childhood education centres received disaster training, these were limited pilot projects. In one flood-prone subdistrict, a programme training preschool teachers in flood emergency drills dramatically improved their knowledge and confidence (participant preparedness satisfaction rose to 89%, a 29% increase) (Efastri, Suharni & Zaputra 2024). However, such initiatives were not yet widespread. Overall, pre-disaster mitigation for child health was only partially implemented – plans and guidelines existed, but many had not been fully translated into community-level action. This shortfall set the stage for difficulties once the floods and landslides occurred.

### Emergency response: Addressing children’s health needs

When the floods and landslides struck in late November 2025, the immediate emergency response faced overwhelming challenges. In the critical first hours and days, local and national responders mobilised search and rescue teams, established temporary shelters and delivered relief supplies by any means possible – even resorting to helicopter air drops for isolated communities. The Disaster Response Task Force of the Indonesian Pediatric Society responded to the disaster by deploying volunteer teams across three provinces – Aceh, North Sumatra and West Sumatra (Badan Nasional Penanggulangan Bencana 2025). Approximately 200 paediatric specialists were mobilised to provide medical assistance to affected children in the disaster areas. In addition to delivering medical care, the Indonesian Pediatric Society’s Disaster Response Task Force also provided logistical support, including approximately 60 water tanker trucks supplying clean water, as well as rice, cooking oil, clothing and food supplies for victims of the floods and landslides in Sumatra.

Despite various efforts being undertaken, the magnitude of the disaster and the extensive damage to infrastructure significantly hampered coordination during the early stages of the response. In the first week, multiple challenges emerged. Among them were severely disrupted communication access because of damaged infrastructure, overlapping and unclear coordination mechanisms, and a highly distressed population that had lost homes and possessions and was in urgent need of assistance. Road access was also compromised by landslides and flooding, severely obstructing the delivery of both logistical and medical aid (Badan Nasional Penanggulangan Bencana 2025; Marzun et al. 2025). Several affected districts reported confusion regarding command structures and information flow, reflecting a lack of adequate preparedness drills. This confusion gradually subsided as national coordination improved. However, this situation provides a critical lesson: That intersectoral collaboration training as part of disaster mitigation can reduce early-phase chaos during emergency response operations. Public communication was also found to be suboptimal. Discrepancies between official updates and conditions on the ground created frustration among survivors. These issues illustrate that the lack of trained activation of the Incident Command System (ICS) and weak inter-agency integration can delay the delivery of critical services especially for children in the immediate aftermath of a disaster.

Within just a few days, it became evident that children were experiencing a wide range of health problems in the flood-affected zones. Overcrowded emergency shelters and even open-air camps exposed displaced families to environmental hazards and unsanitary conditions. Many children were residing in makeshift tents or under tarpaulins with minimal protection from rain and mosquito exposure, rendering them highly vulnerable to the elements and vector-borne diseases. Unsurprisingly, field health teams reported a surge in common post-disaster illnesses such as upper respiratory tract infections (coughs and colds), diarrhoeal diseases and skin infections including scabies and dermatitis. Infants were particularly at high risk because of the damp, unhygienic environment, with aid workers voicing concern over inadequate shelter conditions lacking warmth and sanitation, thus increasing the risk of hypothermia and infection. Although some trauma-related injuries were reported, infectious diseases and environmental health threats quickly became the dominant concern for the paediatric population. Children experienced a range of health problems, as illustrated in Figure 2.

**FIGURE 2:**
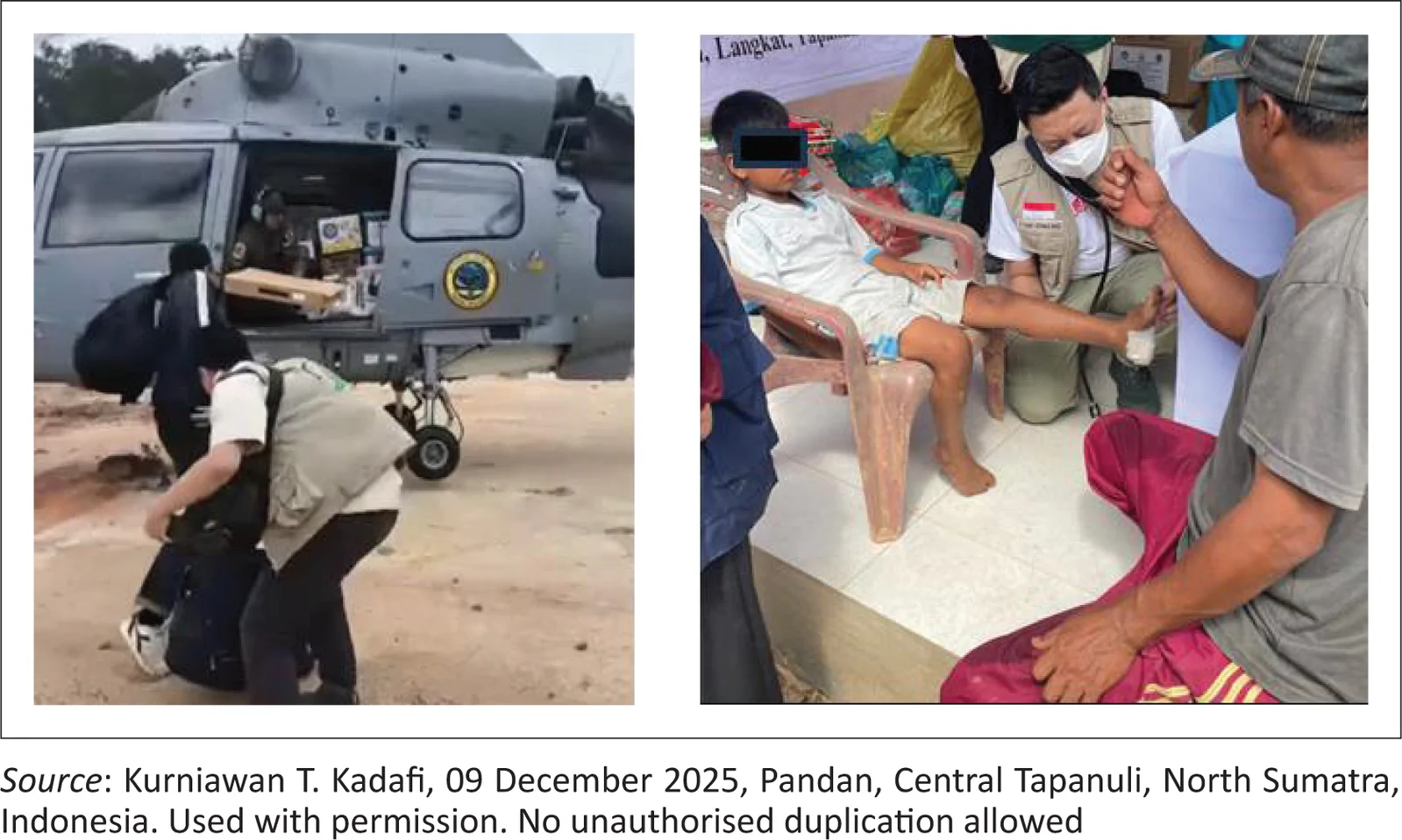
The Disaster Response Task Force of the Indonesian Pediatric Society responded to the 2025 Sumatra floods by deploying volunteer teams to the affected areas.

Emergency medical teams, both from government agencies and NGOs, were deployed to provide basic health services. These teams managed cases of dehydration and fever, distributed essential medicines and administered vaccinations where possible to prevent outbreaks. However, they also faced less visible but equally critical health threats. Children with chronic illnesses, such as epilepsy or type 1 diabetes, suddenly lost access to their routine medications because of road inaccessibility. These children faced life-threatening interruptions in care; for example, a child with diabetes who could not receive insulin because of the disaster was at risk of acute complications. Such scenarios underscore a critical gap in disaster preparedness planning: ensuring continuity of care for children with special health needs. In Sumatra, ad hoc solutions such as airlifting critical patients and requesting emergency supplies of essential medicines were implemented, but not all cases could be addressed in time. This experience underscores the necessity of integrating chronic paediatric care into contingency plans, including maintaining registries of medically fragile children and prepositioning paediatric medicines in disaster-prone areas (Iyer et al. 2025; Lindsay & Hsu 2024; Newman & Leochico 2022; Suciana, Agustiningrum & Pramono 2025). The isolation of flood- and landslide-affected areas required healthcare workers to travel on foot to reach affected communities and provide healthcare services, as illustrated in Figure 3.

**FIGURE 3:**
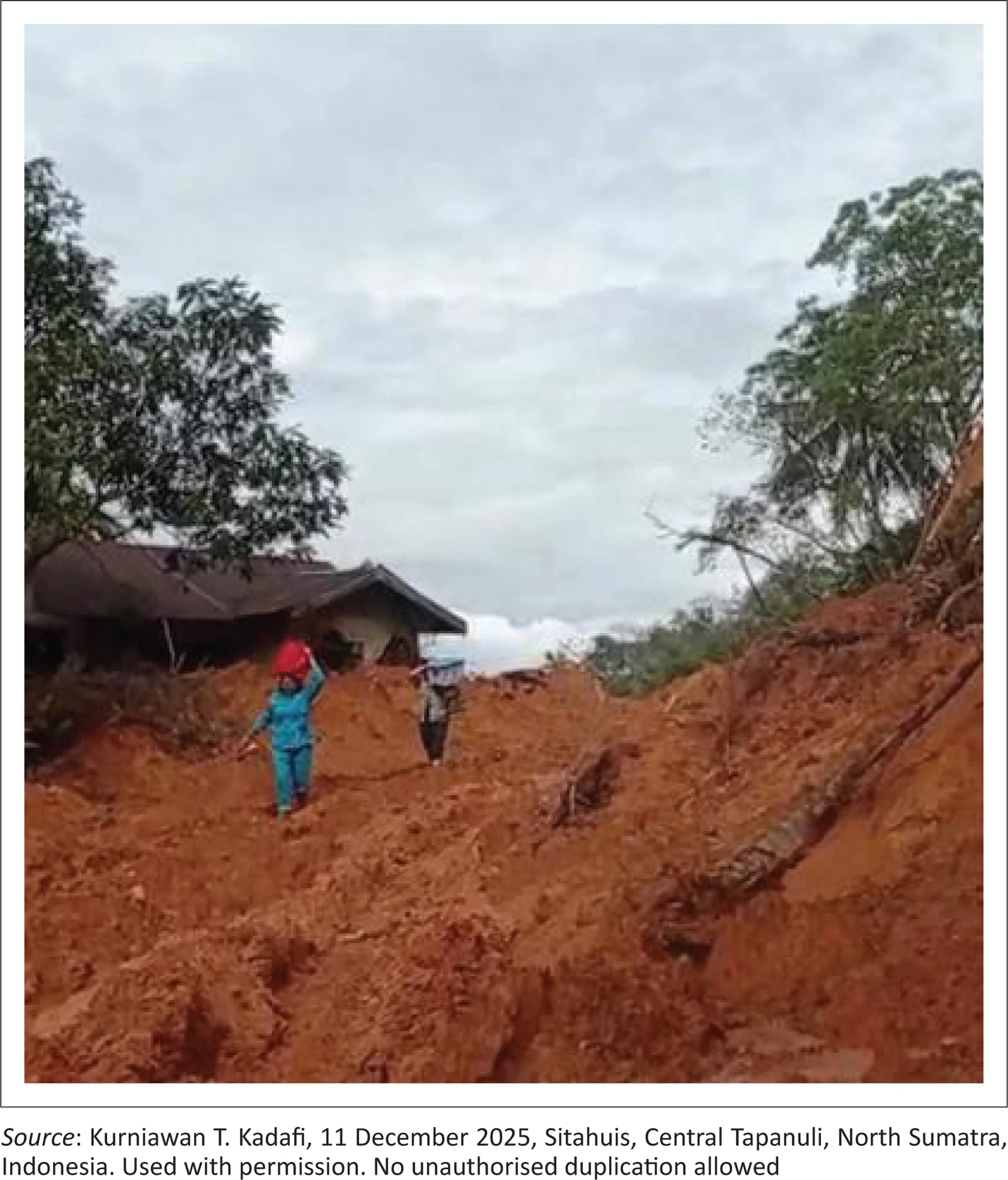
The mobile clinics faced extremely difficult terrain, as teams had to walk through severely damaged landslide-affected areas.

Beyond physical health, the psychosocial impact on children required urgent attention. Many youngsters had witnessed terrifying scenes – their homes collapsing or being swept away – and some had lost family members. Humanitarian organisations on the ground set up child-friendly spaces and psychosocial support programmes almost immediately to help children cope. For example, Save the Children, working with local partners, established safe play areas where children could draw, sing and play under supervision (UN OCHA IHCP 2025). These activities were designed to restore a sense of normalcy and provide psychological first aid. Trained counsellors and volunteers engaged children in group games and storytelling, allowing them to express fears and begin healing. Such interventions are vital: The psychological toll of disasters on children (acute stress, anxiety, nightmares and potential post-traumatic stress disorder) can be long-lasting if not addressed. Indeed, mental health support for affected children in Sumatra was as important as treating their physical ailments (Bhadra 2022; Parrott et al. 2025).

Despite the highly challenging conditions, the joint response by government agencies, NGOs and community groups yielded positive outcomes over time. Mobile clinics and health posts successfully reached many displaced populations. The Provincial Health Office, supported by international agencies, conducted health screenings for children within the first few weeks. Emergency immunisation campaigns were also launched in displacement camps to prevent measles and other communicable diseases. The Disaster Response Task Force of the Indonesian Pediatric Society was deployed to the Aceh, North Sumatera and West Sumatera region, strengthening clinical capacity, including the provision of paediatric services in remote areas (UN OCHA IHCP 2025).

Nevertheless, the disaster response in Sumatra highlighted several critical areas requiring improvement. Coordination mechanisms must be strengthened, and enhanced communication and data sharing between national and local levels have the potential to reduce delays and misinformation. Most importantly, the response underscored the necessity of recognising children as a distinct priority group. They require targeted services such as child-friendly shelters, paediatric-specific formulations of medications and family tracing for separated minors – rather than a one-size-fits-all approach applied across age groups. In disaster settings, children’s survival depends on the recognition of their unique needs and the tailoring of interventions accordingly, with child protection upheld as a humanitarian imperative, not treated as an afterthought.

### Post-disaster recovery and building resilience for children

In the aftermath of the acute emergency, Sumatra moved into a protracted recovery phase. The floods and landslides left a trail of devastation that would take months or years to repair: Entire villages wiped off the map, thousands of homes destroyed and infrastructure (roads, bridges, schools and health centres) in ruins (Famvin 2025). For children, the recovery period is a critical time that can greatly influence their long-term well-being and development (Zacher et al. 2022). In the Sumatra disaster, many children remained in evacuation centres or temporary shelters for extended periods because their communities had been obliterated. Living in crowded camps for weeks to months posed significant health risks. Communicable diseases thrive in such conditions, there were serious concerns about tuberculosis, diphtheria and other infections spreading among displaced families (Kadafi et al. 2022, 2023). Humanitarian health agencies intensified disease surveillance, and the Ministry of Health, supported by the Indonesian Pediatric Society, carried out mass immunisation campaigns (such as diphtheria-tetanus vaccination) to mitigate this threat. Providing access to clean water and sanitation in the camps was also a priority to prevent diarrhoeal outbreaks. Recovery plans for Sumatra prioritised rebuilding vital infrastructure such as water supply systems and clinics to restore basic public health functions.

Psychologically, the long-term impact on children was profound. Many children not only experienced the traumatic disaster itself but were also forced to live with prolonged uncertainty and instability in its aftermath. The loss of homes and possessions coupled with witnessing their parents’ struggles during the recovery process placed a substantial emotional burden on young survivors (Musa et al. 2014; Zacher et al. 2022). Children often internalise the stress experienced by their caregivers (Zacher et al. 2022). In Sumatra, observers reported that children and adolescents exhibited anxiety and sadness after witnessing their families’ distress and the destruction of their communities. Accordingly, mental health and psychosocial support (MHPSS) remained a critical priority throughout the recovery phase. A range of community-based interventions was subsequently implemented, including structured group counselling sessions and peer-support activities within temporary housing sites, aimed at promoting emotional recovery and restoring a sense of safety and normalcy among affected children (Marthoenis 2025).

Schools play a stabilising role for children following disasters; however, in this case, hundreds of schools were damaged or destroyed by flooding. As a result, approximately 270 421 students across the provinces of Aceh, North Sumatra and West Sumatra experienced disruptions to their education because of damage to 3098 schools. To address this situation, the government, in collaboration with NGOs, established temporary learning spaces (such as tents or makeshift classrooms) and provided educational materials to enable children to resume schooling while reconstruction efforts were still ongoing. Restoring daily routines such as returning to school is a key component in helping children regain a sense of normalcy and fostering hope for the future (UN OCHA IHCP 2025).

A guiding principle of the recovery phase is that the government must ‘be present’ for affected populations, particularly children. In Indonesia, authorities acknowledged the necessity of a strong state presence during the recovery process in Sumatra, while also recognising that the scale and complexity of the disaster exceeded the capacity of government action alone. Consequently, a coordinated, multisectoral approach was required. In practical terms, this involved forging partnerships in which local and national authorities worked closely with community-based organisations, professional associations (such as the Indonesian Pediatric Society) and NGOs to address the complex and interrelated post-disaster challenges faced by children. For example, child protection agencies collaborated with health and education sectors to ensure the continuity of care, safeguard children from exploitation and neglect and support their physical, psychological and social recovery in the aftermath of the disaster. For example, child protection agencies collaborated with law enforcement authorities and social workers to establish systems for the identification and support of unaccompanied and orphaned children. Health authorities worked in partnership with international experts to rebuild health facilities with greater resilience to future hazards, in line with the principle of *building back better*. Meanwhile, the Ministry of Social Affairs and the Ministry of Education developed cash assistance programmes and school rehabilitation initiatives, respectively, with the aim of mitigating the long-term impacts of the disaster on child poverty and educational continuity. This spirit of ‘synergy’, a term commonly used in the Indonesian context to denote coordinated and collaborative action, represents a critical element of the post-disaster recovery phase. Effective recovery requires strong governmental leadership to foster cooperation, minimise inter-institutional frictions and align diverse stakeholders towards a shared national interest, with affected communities as the central focus. By pooling resources and expertise, stakeholders sought to ensure that children’s needs including shelter, nutrition, healthcare, psychosocial support and education were addressed in a comprehensive and integrated manner. The Sumatra experience underscores that recovery is not solely about physical reconstruction but also about restoring the social and emotional well-being of the youngest survivors. Only through an inclusive recovery process, with a deliberate focus on children, can disaster-affected communities truly recover and become more resilient to future shocks.

### Implications for disaster risk reduction theory

The Sumatra floods of 2025 underscore important theoretical implications for DRR frameworks, particularly the need to put children at the centre of disaster planning and resilience-building. Traditional DRR models – often grounded in the disaster management cycle of mitigation, preparedness, response and recovery – have tended to treat populations as homogeneous or focus predominantly on adults. However, as Schloemer and Timm (2025) observe, it has been far from standard to explicitly include paediatric considerations in each phase of the cycle (Schloemer & Timm 2025). Children constitute a significant share of the population and have unique vulnerabilities in disasters, yet DRR theory and plans frequently overlook these differences. The implication is that DRR frameworks must evolve to become more child-centred and inclusive. This means recognising that risk factors and protective factors for children can differ from those of adults (e.g. children may be more physically susceptible to certain hazards and psychologically affected in distinct ways (Chiu et al. 2022).

One theoretical advancement highlighted by this case is the concept of child-centred DRR. This approach, which is increasingly advocated in international forums, posits that children are not only beneficiaries of protection but can also be active participants in reducing disaster risk. In Indonesia’s context, the contingency planning initiative involving children and youth demonstrates this idea: Planning that respects children’s rights and involves them in drills and decision-making leads to more effective outcomes (Mitchell et al. 2008). It aligns with broader theories of community-based DRR and resilience, which hold that empowering all segments of society (including youth) creates a more robust defence against disasters. The experience in Sumatra demonstrates that when children have not received preparedness education and emergency simulation training, even when such initiatives exist only as limited pilot programmes, they face greater difficulties in coping with disasters, potentially compromising life-saving responses. This provides theoretical support for incorporating age-appropriate DRR education (such as school-based preparedness curricula and evacuation drills for children) as a staple of disaster mitigation strategies.

Another theoretical implication is the need to integrate health and protection perspectives into the core of DRR theory. The Sendai Framework for Disaster Risk Reduction 2015–2030 emphasises that disaster plans should account for vulnerable groups and critical services, including health and education. The Sumatra experience reinforces this by illustrating how the breakdown of health systems and schooling exacerbated the disaster’s impact on children. It suggests that resilience theory must encompass the resilience of health and social systems that cater to children. For instance, a resilient community is one where hospitals, clinics and schools are built or retrofitted to withstand hazards, and where contingency plans ensure continuity of healthcare and education for children post-disaster. The notion of resilience also extends to psychological resilience theoretical models of disaster resilience that should include metrics or considerations for mental health recovery of children, not just physical reconstruction. In summary, the lessons from Sumatra support theoretical frameworks that are multidisciplinary and child-inclusive, merging insights from paediatric public health, developmental psychology and DRR. By refining theory to fully account for children’s needs and capacities, we can ‘complete the disaster cycle’ for children, ensuring no phase, from mitigation to recovery – leaves them behind (Schloemer & Timm 2025).

### Implications for emergency response practice

From an operational perspective, the Sumatra disaster provides clear lessons for emergency response practice, particularly with regard to health emergency teams and field coordination for child health services. Firstly, the disaster underscores the importance of countries having well-trained EMTs that include specialists with paediatric and neonatal expertise. The World Health Organization’s EMT initiative encourages countries worldwide to develop teams that meet minimum standards and are self-sufficient during disasters. A recurring challenge in many low- and middle-income countries is that healthcare providers often have limited experience in paediatric disaster medicine, including adapting triage and clinical management to children’s needs or determining appropriate paediatric medication dosing under field conditions. Moving forward, practical training programmes must explicitly address vulnerable populations such as children: Mass-casualty simulations should include paediatric victims, EMT curricula should incorporate common paediatric emergency scenarios, and medical stockpiles should include child-sized equipment, ranging from needles to oxygen masks (Khorram-Manesh 2025).

Another key practical implication is the need to strengthen coordination mechanisms to prevent the early-stage chaos observed in several areas during the Sumatra flood response. The early implementation of an ICS and health cluster coordination can substantially improve efficiency (Sartipi et al. 2025). In practice, this requires regular multiagency drills in which Indonesia’s National Disaster Management Agency, health workers, the military and NGOs train within a unified response structure. The Sumatra case demonstrated that limited familiarity with joint operations contributed to communication breakdowns. Health emergency teams deployed to the field require clearly defined roles such as who manages the field hospital, how patient referrals between EMTs and local hospitals are organised, and how disease surveillance data are shared. The WHO recommends establishing an EMT Coordination Cell (EMTCC) within the national Emergency Operations Center to integrate domestic and international medical teams during large-scale disasters (Melkonyan et al. 2025). Indonesia could institutionalise this practice nationally. By doing so, when multiple EMTs – including those from NGOs or other provinces – are mobilised, they can be deployed efficiently to areas of greatest need, particularly for children and other vulnerable groups. Clear decision-making regarding the activation of regional or government hospitals and the deployment of Type 2 or Type 3 EMTs to disaster sites is also critical to accelerating health service delivery (Kaim et al. 2023, 2025).

Equally important, MHPSS must be treated as a standard component of emergency health response, rather than as an optional add-on. The Sumatra disaster highlighted the crucial role of child-friendly spaces and counselling services during the acute phase. Many EMTs have traditionally focused on physical trauma and infectious diseases; however, emergency response practice must broaden to include mental health professionals or, at a minimum, equip frontline responders with training in psychological first aid. Simple measures such as establishing safe play areas, maintaining daily routines for children and providing reassurance can substantially reduce trauma in chaotic emergency environments. The global humanitarian community increasingly recognises MHPSS as a core element of health response, and the Sumatra experience reinforces that, for children’s recovery, such support must begin immediately during the response phase (Alghamdi 2022; Simon & Al-Ghailani 2024).

Finally, disaster response practice must ensure the continuity of essential child health services. This includes maintaining immunisation coverage to prevent deadly outbreaks among displaced children and ensuring uninterrupted care for those with chronic conditions. Emergency health responders should have protocols to identify children with special health needs and to secure access to essential medications or therapies through emergency procurement or evacuation when necessary. In Sumatra, the lives of some children with chronic illnesses were placed at risk because such systems were not fully operational. The Disaster Task Force of the Indonesian Pediatric Society documented cases in which parents of children with epilepsy reported seizures occurring because of difficulties accessing hospitals after road networks were severed; task force members intervened to assist affected children under these conditions. Going forward, practices such as establishing referral mechanisms for paediatric subspecialty care during disasters and integrating chronic disease management into emergency medical plans are essential.

### Implications for policy and governance

At the policy level, the Sumatra floods highlighted the need for stronger governance frameworks that formally integrate child protection and child health into disaster management. Policymakers should draw on these lessons to ensure that future disasters are met with a more prepared and coordinated response for children. One key implication is that national and local disaster policies must explicitly prioritise children. This could take the form of including child-focused provisions in disaster management laws and plans – for instance, mandating that every district’s DRR plan has a section on safeguarding children (covering everything from evacuation procedures for schools to stockpiling paediatric medicines) (Lassa et al. 2023). Indonesia’s existing disaster law framework can be augmented by incorporating standards from international agreements such as the Sendai Framework and the *Global Framework for Climate Services* with regard to children. The fact that multiple ministries (Health, Education, Women’s Empowerment and Child Protection, Social Affairs, etc.) were involved in the contingency planning for children is a positive sign; policy should institutionalise such multisector collaboration. For example, a policy could establish a standing inter-ministerial committee on Children and Disasters, ensuring that in peacetime, these agencies jointly prepare, and during crises, they jointly respond in a coordinated way.

Another crucial policy implication is investing in resilient infrastructure and systems that service children. Governments should enforce building standards that make schools and hospitals disaster-resilient. (Indonesia has a Safe Schools initiative which needs continual support and expansion.) Policies that fund the retrofitting of schools or the pre-positioning of portable schooling kits and temporary classrooms can significantly reduce educational disruption after events such as Sumatra. Similarly, health system policies should ensure that every region has a health crisis contingency plan that includes provisions for maternal and child health (for instance, maintaining a reserve of vaccines and therapeutic food for infants). The Sumatra disaster also shed light on the importance of data and information systems. Policy can mandate the development of registries for vulnerable children (such as orphans, those with disabilities, or chronic illnesses) in disaster-prone regions, so that if an emergency occurs, responders can quickly identify and reach those high-risk children. Data-sharing agreements between agencies (like health and social services) during disasters should be established ahead of time to facilitate this.

Policy also needs to strengthen support for community-based and NGO efforts that focus on children. In Sumatra, much of the direct child-focused support – such as the establishment of child-friendly spaces and the distribution of child-specific relief items – was provided by NGOs and local community groups. Governments should recognise these actors as integral partners within formal disaster response frameworks. Doing so would help ensure that, in future disasters, there is no artificial division between governmental and non-governmental efforts; instead, all response activities should be coordinated within an integrated system, particularly for services related to child health.

Finally, the Sumatra disaster case calls for policy to incorporate a feedback and learning mechanism centred on children’s outcomes. After-action reviews (AARs) and evaluations following disasters should include an assessment of how well children’s needs were met. Schloemer and Timm’s concept of a Paediatric After-Action Report is relevant here: It proposes a template of critical questions about paediatric needs in recovery, aimed at improving future preparedness (Schloemer & Timm 2025). Policymakers should mandate that such evaluations be done and that their recommendations inform policy updates. For example, if an AAR finds that children in a certain disaster did not receive timely psychological support, policy could be revised to fund the training of more MHPSS professionals or creation of a national rapid-response psychosocial team for children. In essence, policymaking for DRR should be dynamic and evidence-based, evolving with each event to close gaps.

## Conclusion

The 2025 Sumatra flood and landslide disaster serves as a stark reminder that children demand special attention in every aspect of disaster management. In theory, Indonesia had many of the right elements: Disaster risk reduction frameworks, paediatric-focused guidelines and trained emergency teams. In reality, however, the execution of these plans fell short of fully safeguarding children’s health and well-being amidst the chaos. Many child victims suffered common post-disaster illnesses, trauma and disruptions that could have been mitigated with more effective preparation and coordination. Bridging this gap between ‘ideal vs. reality’ requires concerted efforts on multiple fronts.

Theoretically, we must enrich disaster models to consistently include children’s needs – viewing DRR and resilience through a child-centred lens so that strategies are inclusive and equitable. Practically, emergency response organisations and medical teams should institutionalise paediatric expertise, rigorous coordination drills and integrated mental health support as standard practice. And at the policy level, governments need to institutionalise child protection in disaster protocols, invest in resilient infrastructure for essential services and foster multisector partnerships, all backed by clear mandates and resources. The Sumatra disaster’s painful experiences have yielded valuable lessons. Implementing those lessons – through improved theory, practice and policy – will not only better protect Indonesian children in future disasters, but also contribute to global knowledge on how to create safer, more resilient environments for the youngest members of society. In disaster risk studies and humanitarian action alike, the message is clear: Protecting children is fundamental to building disaster-resilient communities. When the next disaster strikes, let us ensure that the systems in place are truly ready to shield children from harm, help them recover quickly and allow them to continue growing and thriving despite adversity.
